# Simultaneous molecular MRI of extracellular matrix collagen and inflammatory activity to predict abdominal aortic aneurysm rupture

**DOI:** 10.1038/s41598-020-71817-x

**Published:** 2020-09-16

**Authors:** Lisa C. Adams, Julia Brangsch, Carolin Reimann, Jan O. Kaufmann, Rebecca Buchholz, Uwe Karst, Rene M. Botnar, Bernd Hamm, Marcus R. Makowski

**Affiliations:** 1 Charité –Universitätsmedizin Berlin, corporate member of Freie Universität Berlin, Humboldt-Universität Zu Berlin, and Berlin Institute of Health, Charitéplatz 1, 10117 Berlin, Germany; 2 Department of Veterinary Medicine, Institute of Animal Welfare, Animal Behavior and Laboratory Animal Science, Freie Universität Berlin, Königsweg 67, Building 21, 14163 Berlin, Germany; 3 Division 1.5 Protein Analysis, Federal Institute for Materials Research and Testing (BAM), Richard-Willstätter-Str. 11, 12489 Berlin, Germany; 4 Department of Chemistry, Humboldt-Universität zu Berlin, Brook-Taylor-Str. 2, 12489 Berlin, Germany; 5 Institute of Inorganic and Analytical Chemistry, Westfälische Wilhelms-Universität Münster, Corrensstr. 30, 48149 Münster, Germany; 6 School of Biomedical Engineering and Imaging Sciences, King’s College London, St Thomas’ Hospital Westminster Bridge Road, London, SE1 7EH UK; 7 Wellcome Trust/EPSRC Centre for Medical Engineering, King’s College London, London, UK; 8 BHF Centre of Excellence, King’s College London, Denmark Hill Campus, 125 Coldharbour Lane, London, SE5 9NU UK; 9 Escuela de Ingeniería, Pontificia Universidad Católica de Chile, Santiago, Chile; 10 School of Medicine, Department of Diagnostic and Interventional Radiology, Technical University of Munich, 81675 Munich, Germany

**Keywords:** Medical research, Biomarkers, Prognostic markers

## Abstract

Abdominal aortic aneurysm (AAA) is a life-threatening vascular disease with an up to 80% mortality in case of rupture. Current biomarkers fail to account for size-independent risk of rupture. By combining the information of different molecular probes, multi-target molecular MRI holds the potential to enable individual characterization of AAA. In this experimental study, we aimed to examine the feasibility of simultaneous imaging of extracellular collagen and inflammation for size-independent prediction of risk of rupture in murine AAA. The study design consisted of: (1) A outcome-based longitudinal study with imaging performed once after one week with follow-up and death as the end-point for assessment of rupture risk. (2) A week-by-week study for the characterization of AAA development with imaging after 1, 2, 3 and 4 weeks. For both studies, the animals were administered a type 1 collagen-targeted gadolinium-based probe (surrogate marker for extracellular matrix (ECM) remodeling) and an iron oxide-based probe (surrogate marker for inflammatory activity), in one imaging session. In vivo measurements of collagen and iron oxide probes showed a significant correlation with ex vivo histology (p < 0.001) and also corresponded well to inductively-coupled plasma-mass spectrometry and laser-ablation inductively-coupled plasma mass spectrometry. Combined evaluation of collagen-related ECM remodeling and inflammatory activity was the most accurate predictor for AAA rupture (sensitivity 80%, specificity 100%, area under the curve 0.85), being superior to information from the individual probes alone. Our study supports the feasibility of a simultaneous assessment of collagen-related extracellular matrix remodeling and inflammatory activity in a murine model of AAA.

## Introduction

Abdominal aortic aneurysm (AAA) is a life-threatening vascular disease with an up to 80% mortality in case of rupture^1^. The extracellular matrix (ECM) of the tunica media with collagen and elastin as main components is the most important structural component of the aortic wall^2^. AAA formation results from proteolytic imbalance, including ECM matrix remodeling and a progressive weakening of the arterial wall^3^. With regard to collagen, it was previously shown that its degradation in combination with dysfunctional disposition was associated with the onset, progression and rupture of AAA^2,4,5^.

Independent of pathogenesis, the most widely accepted definition of an AAA is based on the diameter of the patients’ aorta exceeding 30 mm^6^. As intervention can be associated with risks, elective aortic repair is not recommended until the risk of rupture exceeds the risk associated with intervention^4^. However, the use of diameter as the primary criterion for intervention fails to fully capture the individual risk, as aneurysms of similar size can significantly vary in progression rate and rupture risk, while there is a scarcity of longitudinal data.

It is in the nature of AAAs, that harvesting tissue in the formative phase is not a practical option. Therefore, we are restricted to the descriptive pathology of advanced AAA acquired during the time of surgical repair^7^. Obviously, such advanced AAAs provide limited insight into the preceding pathophysiological development^7^. The use of animal models is hence of particular importance. Over the past years, dissecting murine AAAs have become a valuable and popular model to study both focal aortic dissection and expansion as well as intramural thrombus formation^8^. While Angiotensin II (AngII)—induced dissecting aneurysms reproduce several important clinical features of human AAAs, including atherosclerosis, ECM remodeling and inflammation as well as an enhanced propensity for the development of AAAs in male mice, there are also differences compared to human AAAs^8^. Other than human AAAs, mural dissecting AAAs have a suprarenal location, develop within days, show a focal medial tear instead of circumferential medial degradation and form an intramural hematoma by adventitial dissection instead of an intraluminal thrombus^9,10^. Naturally, these differences have to be taken into account, when interpreting the results of the present study. While none of the currently used animal models fully mirrors human AAAs, we still believe, that animal models of AAA, such as the commonly used AngII-induced mouse model with dissecting AAAs^11^, may allow for intriguing insights into the developments of AAAs, while at the same time allowing for longitudinal study setups.

Given the current absence of any reliable biomarker for the characterization of AAAs and the identification of individuals at high risk for experiencing AAA, the development and testing of new biomarkers is highly desirable. These markers are ideally tested in a longitudinal setup using an animal model. By combining molecular imaging with MR target-specific probes, pathological processes can be detected and characterized in vivo^12,13^.

In the present study, we aimed to investigate the feasibility of a simultaneous dual-probe approach for the visualization of week-by-week changes of dissecting AAA in an AngII-induced mouse model of dissecting AAAs. A collagen-specific probe served as a marker of collagen matrix degradation, while iron oxide particles were used for the visualization of macrophage infiltration/inflammatory activity. The potential of this de novo multi-target approach was assessed in an outcome-based longitudinal setup.

## Results

**Figure 1:**
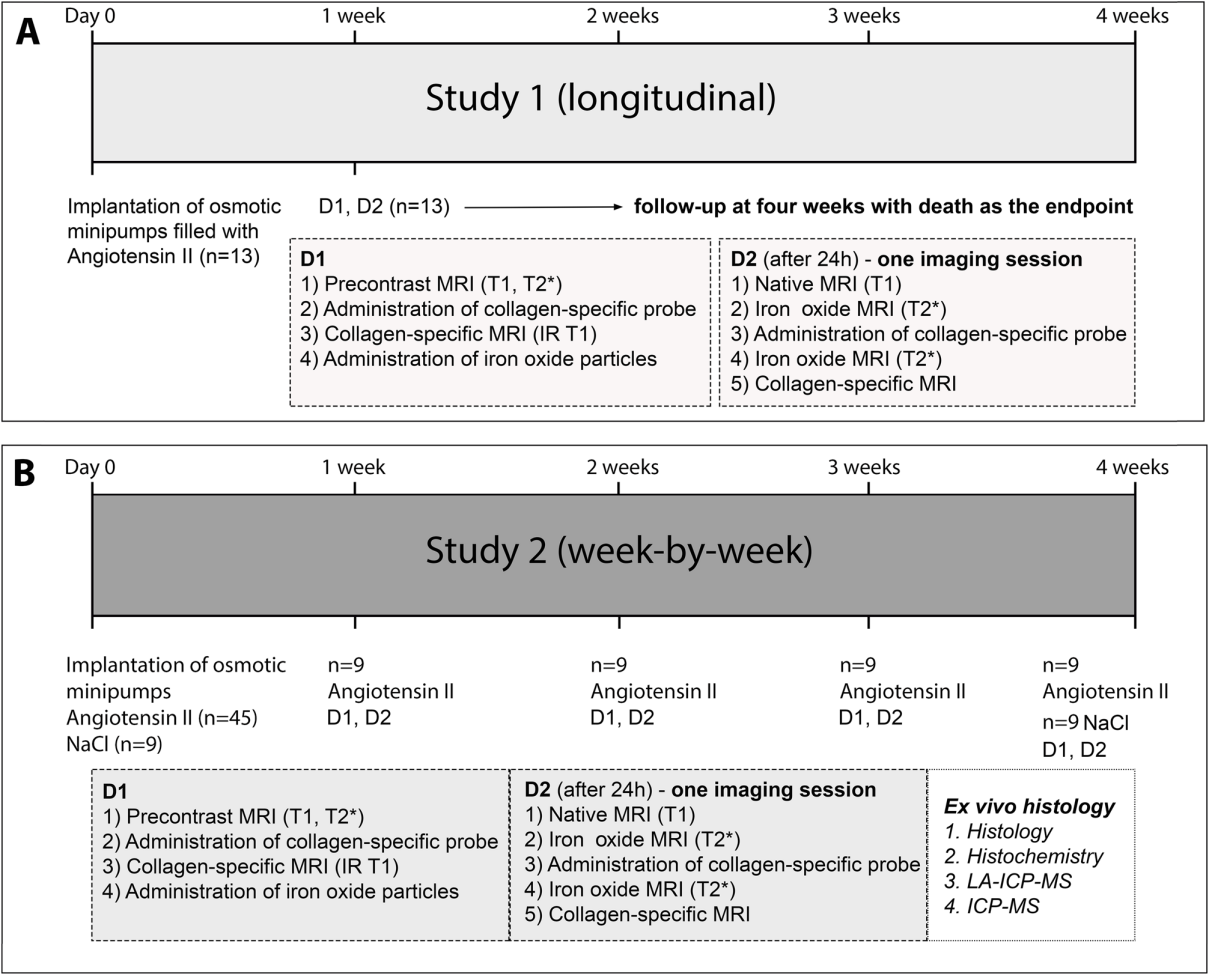
Experimental setup and study design. (**A**) For study 1 (longitudinal), MR imaging was performed during two days after one week (n = 13) with injection of 10 µmol/kg of the applied collagen 1-specific probe (EP-3533) on both days and injection of the inflammation-specific probe (ferumoxytol) at the end of day 1. Animals were then followed up for up to four weeks with death due to aneurysm rupture as the end-point, aiming to evaluate the potential of such a dual-probe approach for the prediction of aneurysm rupture. (**B**) For study 2 (week-by-week), MR imaging was performed during two days according to the same protocol as for study 1 after 1, 2, 3 and 4 weeks of AngII infusion (n = 9 per group). Following the final scan of each week, ex vivo analysis (histology, immunohistochemistry, ICP-MS and LA-ICP-MS) was performed to correlate in vivo with ex vivo results. As control, nine sham-operated wild-type C57BL/6J were implanted with osmotic mini pumps primed with sodium chloride.

**Figure 2:**
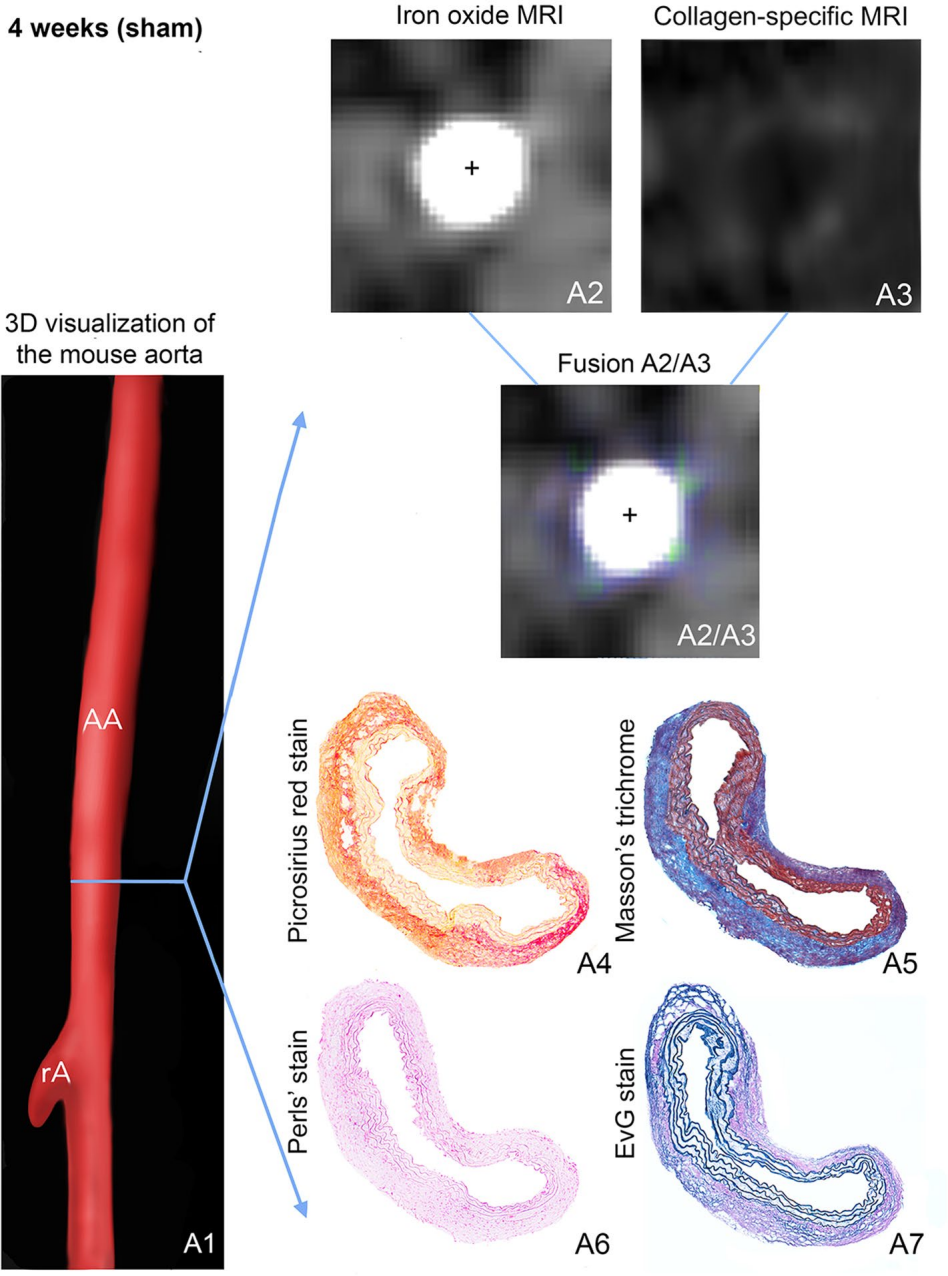
In vivo molecular dual-probe MRI and ex vivo assessment in controls. (**A1**) 3D visualization of the suprarenal abdominal aorta (AA) with the renal arteries (rA) of a male, sham-operated ApoE^−/−^ mouse after 4 weeks of saline infusion. The blue line indicates the orientation of subsequently performed transverse MRI sequences and the corresponding histological sections. Iron-oxide MRI (**A2**) and collagen-targeted MRI (**A3**) show only slight collagen-specific enhancement, while inflammation-associated signal voids are absent. For better visualization of in vivo results, iron oxide MRI and collagen-targeted MRI were fused (**A2**/**A3**), confirming the slight enhancement of the collagen-targeted probe without any negative signal from the inflammation-specific probe. Results of in vivo MR imaging were confirmed by ex vivo histology: Picrosirius red stain (**A4**) and Masson’s trichrome stain (**A5**), both for collagen fibers, show a regular deposition of collagen especially in the adventitial tissue, while the Perls’ stain does not reveal any deposition of inflammation-related iron (**A6**). (**A7**) shows the elastic tissue. *AA* suprarenal abdominal aorta, *rA* right renal artery, ^+^Vascular lumen in arterial TOF.

To determine if the applied collagen-specific probe could reliably characterize week-by-week changes in murine aneurysms, mice were imaged at 1, 2, 3 and 4 weeks prior and following the administration of 10 µmol/kg of the collagen-specific probe. In addition, for the longitudinal setup, 13 mice were also imaged after one week prior and after administering the collagen-specific probe, using death as the endpoint. The study design is visualized in detail in Fig. 1. Sham-operated mice with a continuous saline infusion for a duration of 4 weeks served as controls for the week-by-week study and did not develop dissecting AAAs (refer to Fig. 2).

### ECM collagen and inflammatory activity for prediction of dissecting AAA rupture (longitudinal study 1)

**Figure 3:**
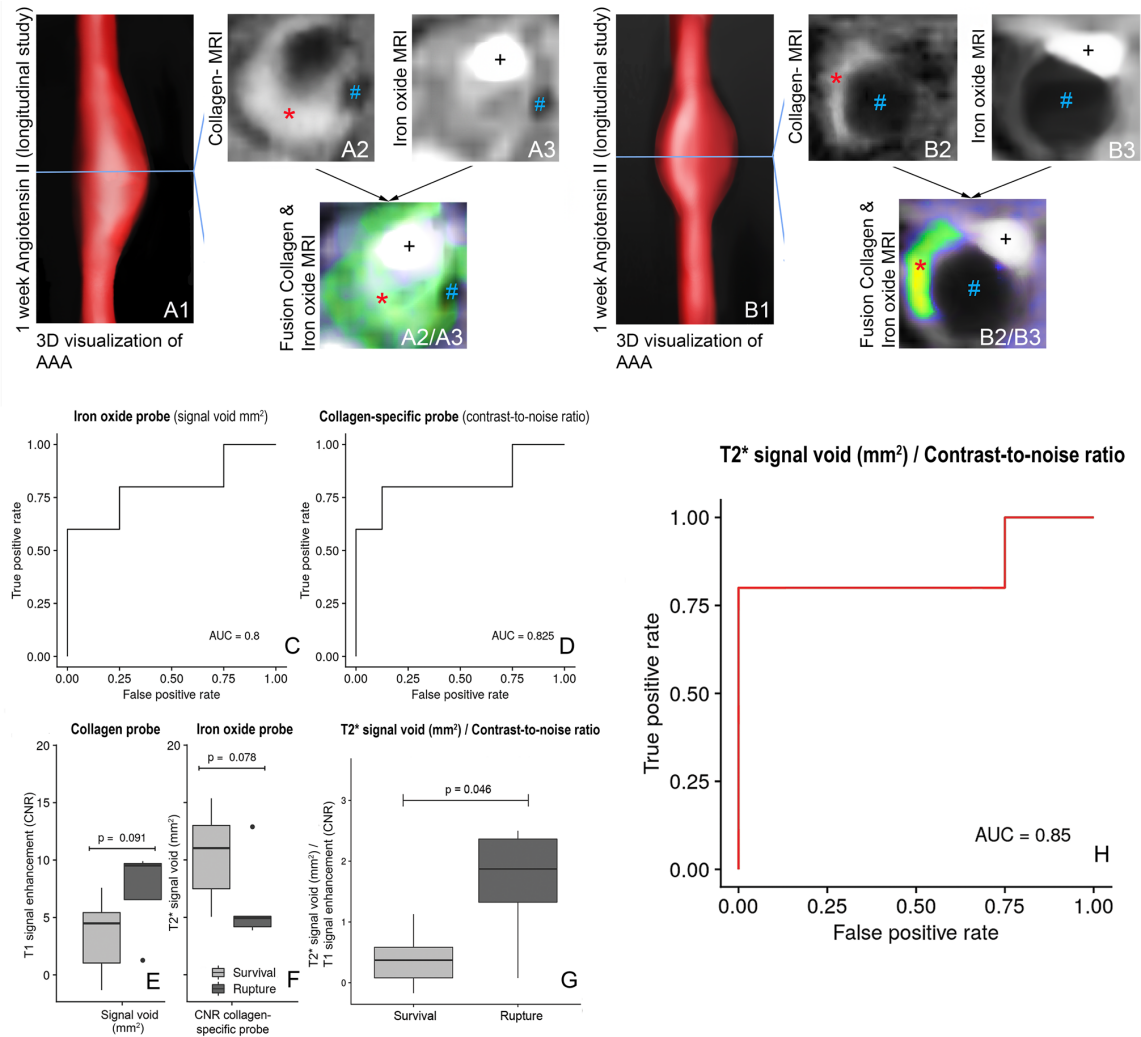
In vivo dual-probe MRI of collagen metabolism and inflammatory activity of a stable and rupturing AAA (longitudinal study). (**A1**,**B1**) 3D visualizations of suprarenal abdominal aortas. (**A2**) Strong enhancement from the collagen-targeted probe in the surviving animal and (**B2**) notably less signal enhancement in the animal suffering a fatal aneurysm rupture. (**A3**) A minor signal void in the surviving animal compared to a larger signal void in the animal suffering a fatal rupture (**B3**). (**A2**/**A3**,**B2**/**B3**) are fusions of (**A2**) and (**A3**) or (**B2**) and (**B3**) for better visualization of the spatial distribution, with the green to yellow colors and the red stars indicating the enhancement from the collagen-specific probe and the blue double crosses indicating the signal voids. (**C**,**D**), ROC curves for the iron oxide probe (signal voids) (**C**) and the collagen-specific probe (**D**). (**E**,**F**) differences in the collagen-specific probe (**E**) and the size of the signal void (**F**) between stable and fatal AAAs. (**G**) For a quotient of the size of the signal void and T1 signal enhancement (CNR), there was a significant difference between surviving animals and mice with rupturing aneurysms (p = 0.046). (**H**) Combined assessment of both probes allowed for the highest diagnostic performance to predict aneurysm rupture, yielding a sensitivity of 80% and a specificity of 100%. *CNR* contrast-to-noise ratio. *Signal from the collagen-binding probe in the aneurysmal wall, ^#^Signal void from the iron oxide particles, *AAA* suprarenal abdominal aortic aneurysm, *rA* renal artery, ^+^Vascular lumen in arterial TOF.

In order to evaluate the potential of a multi-target MRI approach to evaluate the risk of aneurysm rupture, a longitudinal study of ApoE^−/−^ knockouts was performed, herein after referred to as study 1 (n = 13). In the following, the term surviving animals refers to the mice in study 1, whose aneurysms did not rupture for at least four weeks after infusion of AngII. For both the diameter and the vessel area, we did not find any significant difference between animals with aneurysm rupture and surviving animals (p = 0.341 and p = 0.262, also refer to Fig. S1). Following 1 week after implantation of osmotic minipumps and continuous AngII infusion, surviving animals showed a stronger signal enhancement from the collagen-binding probe (n = 8, Fig. 3A1–A6), while the administration of iron oxide particles resulted in a larger signal void in animals with subsequent deadly rupture of dissecting AAA (n = 5), resulting from the iron oxide particles, which indicate a pronounced influx of proinflammatory macrophages (also refer to Fig. 3B1–B6). However, these differences did not reach significance level (p = 0.091 and p = 0.078, Fig. 3C). Based on the combined information from iron oxide and collagen imaging, there was a significant difference between animals with aneurysm rupture and surviving animals (p = 0.046, Fig. 3D). Using the size of the signal void (mm^2^) after administration of iron oxide particles and a cut-off of more than 9.53 mm^2^, dissecting AAA rupture could be predicted with moderate diagnostic accuracy (sensitivity of 60%, specificity of 100%) and an area under the curve (AUC) of 0.8 (see Fig. 3E). This finding indicates, that iron oxide particles could represent a complementary imaging marker to assess the rupture risk in dissecting AAA. The clearance of iron oxide particles by reticuloendothelial system macrophages changes the magnetic properties of the macrophages, so that they become visible as signal voids^13^.

**Figure 4:**
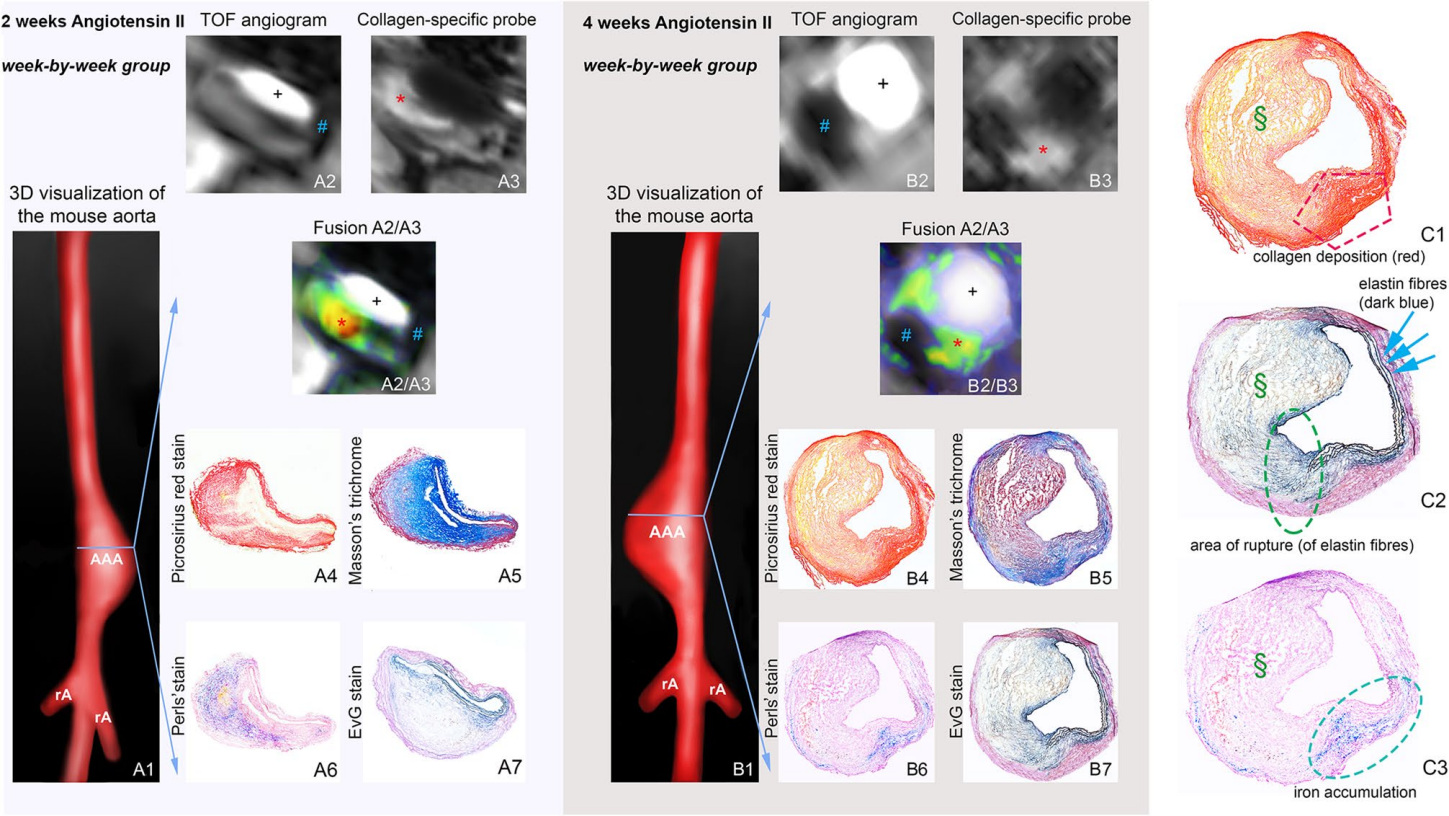
In vivo molecula r MRI with collage-targeted and inflammation-specific probes (week-by-week study). 3D visualization of AAAs after 2 weeks (**A1**) and 4 weeks (**B1**) of Angiotensin II (AngII) infusion. (**A2**,**B2**) Iron oxide MRI indicating signal voids of different sizes for 2-week (**A2**) and 4-week (**B2**) AAAs. (**A3**,**B3**) IR T1-weighted sequences showing areas of intermediate signal enhancement after 2 weeks (**A3**) and four weeks (**B3**) of AngII infusion. **A2**/**A3**) and **B2**/**B3**) are fusions of (**A2**/**A3**) and (**B2**/**B3**), showing both the positive signal of the collagen-targeted probe (green to yellow/orange, with brighter colors corresponding to higher enhancement) and the signal voids of the iron oxide probe. (**A4–7**,**B4–7**): Ex vivo histological measurements using Picrosirius red and Masson’s trichrome for visualization of collagen fibers and Perls’ staining for detection of inflammation-associated iron confirmed the in vivo findings. (**C1**–**C3**) are magnifications of (**B4**,**B7** and **B6**): (**C1**) shows compensatory collagen deposition in the aneurysmal wall after rupture. (**C2**) shows the rupture site (green circle) with the ruptured elastin fibers. (**C3**) shows the iron accumulation at the rupture site. *Signal from the collagen-binding probe in the aneurysmal wall, ^#^Signal void from the iron oxide particles, ^§^Thrombus area. *AAA* suprarenal abdominal aortic aneurysm, *rA* renal artery, ^+^Vascular lumen in arterial TOF.

Regarding the signal of the collagen-specific probe after one week of AngII infusion, animals with subsequent deadly rupture of dissecting AAA (n = 5) demonstrated a lower CNR compared to surviving animals (n = 8), indicating a pronounced breakdown of collagen fibers without compensatory collagen metabolism (also refer to Figs. 3 and 4). Using the signal of the collagen-specific probe and a cut-off value (CNR) less than 5.09, dissecting AAA rupture could be predicted with a sensitivity of 80%, a specificity of 87.5% and an AUC of 0.83 (Fig. 3F).

The highest diagnostic accuracy for assessing risk of rupture in untreated murine dissecting AAAs was achieved by a combined evaluation of the CNR from the collagen-specific probe and the size of the signal voids from the iron oxide particles as a correlate for inflammation. This dual-probe approach (size of signal void in mm^2^/CNR, cut-off value > 1.32) yielded a sensitivity of 80%, a specificity of 100% and an AUC of 0.85 (Fig. 3G) and was superior to the assessment of the individual parameters.

Assuming that a higher collagen content (with a subsequently higher T1 CNR) is associated with AAA remodeling and enhances the stability of the aneurysm, while the area of the signal void correlates with the degree of active inflammation, this indicates that larger areas of T2* signal voids and lower T1 CNRs might increase the individual risk of AAA rupture, presumably resulting from active inflammation with little remodeling. A quotient also has the advantage, that instead of two parameters, only one combined parameter needs to be considered.

### Dual-probe imaging of extracellular collagen and inflammation for the characterization of dissecting AAA development (week-by-week study 2)

After continuous AngII infusion in ApoE^−/−^ mice, we observed an average increase in the aneurysmal area of 26.8% after one week, of 77.0% after two weeks, of 209.6% after three weeks and of 228.4% after four weeks (also refer to Fig. S2). In earlier-stage aneurysms, up to two weeks after AngII infusion, more aneurysms showed marked in vivo enhancement after administration of the collagen-specific probe, suggesting a collagen-rich response to the degradation of the extracellular matrix, which could be confirmed by ex vivo histology. In this context, histology also revealed an increase in the aneurysm area and the formation of an intramural hematoma in approximately 60% of the cases. Simultaneously, a larger signal void in the area of the aneurysmal wall could be found in T2*-weighted images following the accumulation of iron oxide particles, which could be confirmed by ex vivo histology. We did not observe any calcifications that could have interfered with the detection of iron oxide particles, which was probably also due to the relatively young age of the mice compared to animal models focusing on advanced atherosclerotic lesions. Later-stage aneurysms between three and four weeks after AngII infusion demonstrated extensive remodeling of the arterial wall and often a lower collagen content following collagen degradation without compensatory collagen deposition. This was accompanied by a further increase in the aneurysmal area (Fig. S2). Regarding inflammatory activity, later-stage aneurysms showed a trend for increased inflammatory response with larger signal voids in magnetic resonance imaging, following the clearance of iron oxide particles by reticuloendothelial system macrophages and thus changing the magnetic properties of the macrophages. Also refer to Fig. 4 for examples of aneurysms after two and four weeks after AngII infusion.

### T1-weighted MRI for the evaluation of the gadolinium-based collagen-specific probe

Prior to the injection of the collagen-specific probe, the aortic wall showed a low CNR on all MRI scans in both AngII- and saline-infused mice. Following the injection of the collagen-specific probe we observed a significant increase in CNR. The in vivo observations significantly correlated with ex vivo measurements of collagen content on histological sections using Picrosirius red staining (R = 0.55; p < 0.01). Besides, our findings demonstrated the feasibility of a collagen-targeted probe to differentiate collagen-rich from collagen-poor aneurysms (Fig. S3). We found a significant correlation between the CNR of collagen MRI and Picrosirius red staining (Fig. S4A).

### T2*-weighted MRI for the evaluation of inflammatory processes

Prior to the administration of iron oxide particles, no signal voids could be detected in the vessel area of AngII- or saline-infused mice and the aortic lumen appeared bright and circular on all T2*-weighted images. 24 h after injection of iron oxide particles (ferumoxytol), a significant T2* signal loss could be observed close to the dissection site in the aneurysmal wall of AngII—infused animals (Fig. 4), whereas sham-operated animals demonstrated no significant changes (again refer to Fig. 2). With dissecting AAA progression, the size of the observed signal voids increased, while we found no significant differences regarding the T2* signal reduction within the signal void. Regarding in vivo and ex vivo measurements, we found a strong correlation between the size of T2* signal voids and Prussian blue-stained iron particles (y = 1.8 + 1.3x, R = 0.77, p < 0.05, Fig. S4B).

### Potential effects of the two different probes on each other (collagen-specific probe versus iron oxide particles)

We performed CNR measurements on T1-weighted images before and 24 h after the injection of iron oxide particles (n = 9), whereby a good agreement between measurement on days 1 and 2 became apparent (y = 1.3 + 0.83x, R = 0.78, p < 0.05, 0.82, 95% confidence interval 0.40–0.96, Fig. S5), while no significant differences in measurements were found (p > 0.05). 24 h following the injection of iron oxide particles, T2*-weighted imaging was performed before and after the injection of the collagen-specific probe, whereby a strong correlation between both measurements became apparent [y = 0.84 + 0.94x, R = 0.99, p < 0.0001, ICC = 0.99 (95% confidence interval 0.97–0.99)]. Again, there was no significant difference between both time points (p > 0.05). We observed that the iron accumulation was mainly localized at the beginning of the dissecting aneurysm, close to the dissection site, while the strongest signal from the collagen was located more centrally in the aneurysm.

### Gadolinium concentration by inductively-coupled mass spectrometry

For assessment of the absolute gadolinium concentration in dissecting AAAs, ICP-MS measurements were performed in 15 mice (n = 3 per time point and n = 3 sham-operated animals). A strong correlation of in vivo CNR measurements with ex vivo measured gadolinium concentrations as a surrogate for collagen could be observed (also refer to Fig. S4C). Correspondingly, a strong correlation could be observed between in vivo T2* measurements and ex vivo measurements of iron content based on the Perls’ staining (also refer to Figure S4D).

### Spatial localization with laser ablation-inductively-coupled plasma mass spectrometry

**Figure 5:**
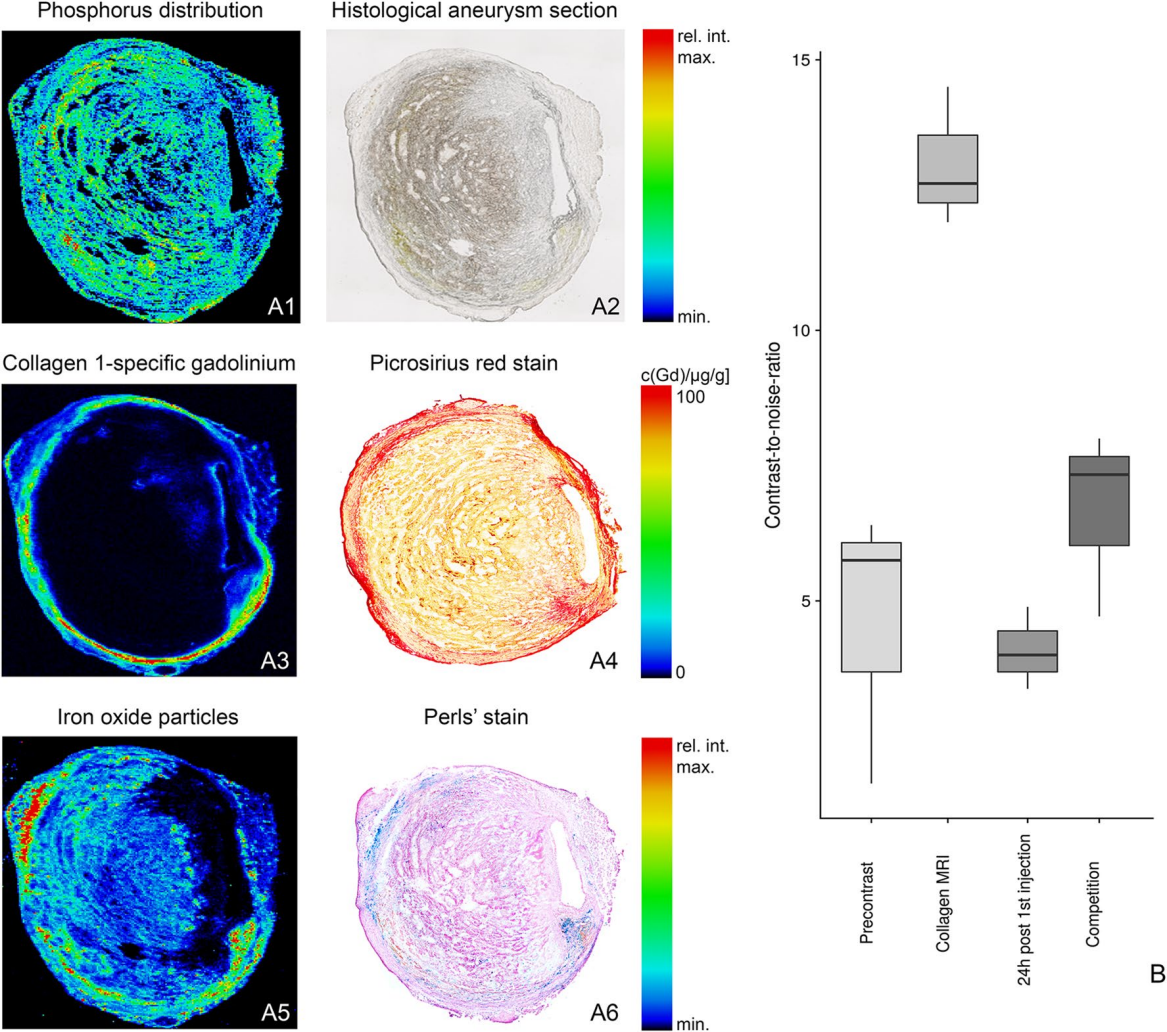
Spatial localization of the collagen 1-specific gadolinium-based probe and iron oxide particles in AAAs using Laser Ablation Inductively Coupled Mass Spectrometry (LA-ICP-MS). (**A1**) For an anatomical overview of the histological AAA section (**A2**), the phosphorus distribution was measured using LA-ICP-MS. (**A3**) LA-ICP-MS confirmed in vivo measurements with the visualization of gadolinium (from the collagen 1-specific gadolinium-based probe) in the aneurysmal wall. A co-localization of gadolinium accumulation with collagen fibers was also demonstrated using Picrosirius red staining (**A4**). (**A5**) LA-ICP-MS furthermore confirmed the accumulation of the iron in the aneurysmal wall. Additionally, the co-localization of iron accumulation was demonstrated with Perls’ Prussian blue staining (**A6**). AAA: abdominal aortic aneurysm. (**B**) In competition experiments, a significant decrease in CNR was found after administration of a collagen peptide (competition) without gadolinium labelling compared to the administration of the gadolinium-based collagen-targeted probe. *CNR* contrast-to-noise ratio.

LA-ICP-MS was used to visualize the spatial distribution of collagen-specific probe-associated gadolinium and iron ions within the aneurysmal wall (Fig. 5A1–6). Dissecting AAA sections of ApoE^-/-^ mice with 1, 2 and 4 weeks of AngII-infusion showed a strong co-localization of targeted gadolinium with red, collagen-positive areas in Picrosirius red staining (Fig. S4). Additionally, a convincing co-localization of iron oxide particles with Prussian blue-positive areas (Perls’ stain) could be demonstrated (also refer to Fig. S4).

### Competition experiments

Specific binding of the collagen probe was shown using an in vivo competition experiment (n = 3) after 4 weeks of AngII infusion. After injection of a tenfold higher dose of a nonparamagnetic collagen peptide, a significant decrease in CNR could be observed compared to the administration of the gadolinium-labeled collagen-specific probe alone (Fig. 5B).

## Discussion

This in vivo study shows, that dual-probe collagen- and inflammation-specific high-resolution MRI can accurately monitor dissecting AAA development and, most importantly, assessment of rupture risk in a longitudinal study. Combining a collagen-specific and an inflammation-specific probe, rupture of murine dissecting AAAs could be predicted with a higher diagnostic accuracy compared to assessing each probe alone. We could demonstrate, that with progression of dissecting AAA, medial rupture occurred with blood infiltrating the adventitial area, resulting in an increase in aortic diameter, whereby in vivo measurements matched ex vivo histological observations.

The hallmark pathology of AAA is a continuing proteolytic imbalance, inducing a degradation of the extracellular matrix as well as a progressive weakening of the arterial wall^3^. The stability of the arterial wall relies on fibrillary collagens in the media and adventitia, whereby an increased turnover of extracellular matrix collagen without adequately matched collagen deposition is considered to be a decisive factor for the rupture of dissecting AAA^14^. This is initiated by members of protease families, which degrade protease-resistant structures of collagen I and III, destabilizing the collagen structure and causing increased collagen degradation combined with inadequate collagen deposition^15^. The second key process is inflammation, which is characterized by migration of macrophages, that secrete inflammatory cytokines, and also by proteases such as matrix metalloproteinases^16^. Both collagen as a marker for extracellular matrix remodeling and macrophages as markers of inflammatory activity thus represent promising biomarkers for the in vivo characterization of abdominal AAAs and the assessment of risk of rupture. Although the two mechanisms are inextricably linked in the pathophysiology of AAA development, as can be seen considering the role of proteases, they still represent two independent processes with changes on a cellular and extracellular level. Formation of AAA becomes most likely in case of an overlap or simultaneous occurrence of both processes. In the present study, we report a reliable diagnostic performance for prediction of AAA rupture for a dual-probe approach based on the combined information of both extracellular remodeling and inflammation, while a single-probe approach produced less convincing results.

While gadolinium-based probes appear as bright signal on T1-weighted MRI due to the T1-shortening effect of gadolinium, iron oxide particles cause signal voids on T2*-weighted imaging, making the MR-based differentiation between the two probes straightforward. It is assumed, that upon entering the bloodstream, injected iron particles are phagocytosed by the reticuloendothelial system and are then taken up by migrating macrophages, inducing signal changes at inflammatory sites due to their magnetic properties^17^. Previous molecular imaging studies with EP-3533 as a collagen-specific imaging probe showed this probe to be sensitive in detecting early stages of fibrosis across different animal and inducement models, demonstrating the robustness of this non-invasive molecular imaging approach to assess collagen content^18,19^. In the present study, histological collagen staining in corresponding sections confirmed the presence of collagen in the areas of T1 signal enhancement.

Animal-based AAA studies will continue to play a key role in gaining insight into the pathophysiology of AAAs, as they are particularly important for studying the early stages of AAAs, because it is not clinically feasible to obtain tissue samples from small human AAAs^20^. The path towards clinical validation of animal findings in humans requires a continues cooperation between preclinical and clinical investigators in order to enable the translation of basic scientific knowledge into diagnostic imaging and possibly medical treatment of AAA. It is, however, important to acknowledge, that some findings from studies in mice cannot be easily and fully translated to human patients due to species-specific differences, including the fact, that murine AAAs develop fast within a few weeks, while human AAAs are a chronic condition, usually developing over the course of years. Previous preclinical and clinical studies investigated the feasibility of imaging inflammation, specifically macrophage activity, in the aneurysmal wall^3,21–23^. Regarding the clinical translation of findings in molecular and cellular imaging, a recent multicenter prospective observational cohort study (“MA3RS”) showed, that a cellular imaging technique based on iron-oxide particle-enhanced MRI may be transferred to humans, allowing for the prediction of clinical events in patients with AAAs^22^. While iron-oxide particle-enhanced MRI could not provide an independent prediction of aneurysm growth of clinical outcomes in a model incorporating known clinical risk factors and it therefore remains to be established, whether clinical outcomes may be improved by new imaging techniques in the future, this still highlights the possibility of translating basic scientific knowledge into clinical practice^22^. A multi-target approach in molecular imaging, such as the one presented in this study, could ideally allow an independent assessment of the risk of rupture and possibly improve clinical outcomes.

Combining the information from collagen remodeling and inflammatory activity by calculating a quotient from the size of the T2* signal void (in mm^2^) and the CNR (T1 signal enhancement), we found that this quotient was significantly different between the surviving animals and the animals with deadly rupture. Correspondingly, simultaneous assessment of both probes also yielded the highest diagnostic accuracy for the prediction of aneurysm rupture. Furthermore, our findings demonstrated the feasibility of a collagen-targeted probe to differentiate collagen-rich from collagen-poor aneurysms, which are more prone to rupture. Using a different collagen-targeted probe (CAN-35 micelle), Klink et al. reported similar results^15^.

With regard to future research possibilities in the field of molecular imaging of AAA, a combination of dynamic contrast-enhanced imaging (DCE) with small iron oxide particles could be promising. DCE-MRI with gadolinium-based contrast agents measures the uptake kinetics in the tissue and can quantify microvascular permeability^24^. For atherosclerotic plaques, Zheng et al*.* recently found that an accumulation of small iron oxide particles was associated with an increased plaque permeability as measured by DCE-MRI, thus supporting prior findings from experimental models linking nanoparticle deposition to impaired endothelial barrier function^24^. For AAA, similar findings could apply. Our findings from a previous study on AAA using an albumin-binding probe already suggested an increased vascular permeability in murine AAA^25^. Another benefit in molecular imaging studies could be obtained by using quantitative MRI protocols, which would not only allow to visualize, but also to quantify the molecular probes used. Especially for the quantification of T1 relaxation times, a reliable sequence [Modified Look Locker Inversion recovery (MOLLI)] has already been developed, which consists of 2 inversion recovery prepared modified Look-Locker experiments (trains) and allows for an estimation of the apparent T1 relaxation time and for the quantification of contrast uptake^26^.

An important limitation of the present study is the difference in anatomy between mice and humans. While murine aneurysms are suprarenal, human aneurysms are usually infrarenal, which may reflect altered mechanical properties due to local differences in collagen and elastin composition^27–29^. In addition, human AAAs are usually not caused by intramural hematoma or dissection. In comparison with other animal models, the present model has the advantage of a spontaneous development of aortic aneurysms after continuous subcutaneous AngII infusion. Also, both probes were administered at a clinical dose and imaging was performed using a clinical 3 T MR scanner. The use of a clinical field strength of 3 T instead of a higher preclinical field strength has several advantages: The effects on T1 and T2 relaxation, rotational correlation and signal properties of molecular probes and potential sources of artifacts (fat, air/tissue interfaces) can vary significantly according to the applied field strength and can therefore limit the direct translation of the findings to human application. Furthermore, we adapted imaging pulse sequences, that are already highly optimized for clinical practice, for preclinical use. Subsequently, the sequences we used can easily be scaled back for clinical practice, greatly facilitating the translation of our protocols from preclinical to clinical use. However, a limitation regarding clinical translation is that the collagen-targeted probe is not approved for clinical use. This also applies to ferumoxytol in the context of AAAs; but other than the collagen-targeted probe, ferumoxytol was approved by the US Food and Drug Administration (FDA) for treatment of iron deficiency anemia in adults with chronic kidney disease, resulting from which its off-label use as a MRI contrast agent has rapidly grown^30^. Finally, the absence of quantitative sequences from the imaging protocol is another potential shortcoming of the present study.

This study shows the potential of simultaneous evaluation of extracellular matrix collagen and inflammatory activity based on a dual-probe molecular MRI approach in a murine model of dissecting AAA. In our setting, the combination of both probes allowed to predict dissecting AAA rupture with a higher diagnostic performance compared to using a classical single-probe approach and enabled in vivo characterization of AAAs.

## Methods

### Animal experiments and study design

All procedures and protocols relating to the animals were carried out in accordance to the guidelines and regulations of the Federation of Laboratory Animal Science Associations (FELASA) and the local Guidelines and Provisions for Implementation of the Animal Welfare Act. The Regional Office for Health and Social Affairs Berlin (LAGeSo) approved this animal study (G 0,169/15).

Male ApoE^−/−^ knockouts (B6.129P2-Apo^Etm1Unc^/J) between the ages of 8 to 12 weeks were obtained from our local Research Institute of Experimental Medicine. Dissecting AAAs were induced by continuous infusion of AngII at a rate of 1000 ng/kg/min for up to 4 weeks through an insertion of osmotic minipumps (Alzet, Model-2004, Durect corporation, Cupertino, CA) into the subcutaneous tissue of the mice^31^.

Figure 1 provides an overview of our study design. Our study consisted of two parts, a longitudinal study (study 1) and a week-by-week-study (study 2).

For study 1 (n = 13), pre- and postcontrast imaging was performed during two days after 1 week before and 30 min after injection of a dose of 10 µmol/kg of the collagen-specific probe (30 µmol/kg gadolinium (Gd) EP-3533) on both days and injection of the inflammation-specific probe (ferumoxytol, 4 mg Fe/kg) at the end of day 1. Imaging on day 2 was performed approximately 24 h after the injection of ferumoxytol. Animals were then followed up over a period of four weeks with death as endpoint. The aim was to evaluate the potential of a dual-probe approach for the prediction of aneurysm rupture. EP-3533 was used as a collagen-specific probe^32,33^ and ferumoxytol was applied for inflammation-specific imaging.

For study 2, MR imaging was performed during two days according to the same protocol as for study 1, but with subsequent tissue harvesting 1, 2, 3 and 4 weeks after AngII infusion (n = 9 per group, total n = 36). This was followed by histology/immunohistochemistry, laser-ablation-inductively-coupled plasma-mass spectrometry (LA-ICP-MS) and inductively-coupled-mass-spectroscopy (ICP-MS). All measurements for the week-by-week study were conducted blinded to the study intervention (AngII infusion versus sham-operated animals) as well as the aneurysm age (1–4 weeks). As controls for study 2, nine sham-operated wild-type C57BL/6J were implanted with osmotic mini pumps primed with sodium chloride.

For the week-by-week study, pre- and postcontrast imaging with subsequent tissue harvesting was performed 1, 2, 3 and 4 weeks after AngII infusion (n = 9 per group, total n = 36). This was followed by histology/immunohistochemistry, laser-ablation-inductively-coupled plasma-mass spectrometry (LA-ICP-MS) and inductively-coupled-mass-spectroscopy (ICP-MS). All measurements for the week-by-week study were conducted blinded to the study intervention (AngII infusion versus sham-operated animals) as well as the aneurysm age (1–4 weeks).

### MR imaging and analysis

Imaging was performed at a 3 T Siemens MRI scanner (Biograph-mMR, Siemens-Healthcare-Solutions, Germany) with a clinically approved single loop coil (47 mm, Siemens Healthcare Solutions, Erlangen, Germany) in supine position. To prevent rapid cooling during the imaging, the body temperature (37 °C) of the mice was monitored with an MR-compatible heating system (Model 1,025, SA Instruments Inc, Stony Brook, NY). The imaging protocol included: A low-resolution three-dimensional gradient echo scout scan, a two-dimensional time-of-flight angiography (2D-TOF) in transverse plane (matrix = 960, field of view (FOV) = 200 mm, 40 slices, slice thickness = 0.35 mm, repetition time (TR) = 35 ms, echo time (TE) = 4.4 ms, spatial resolution of 0.2 × 0.2 × 0.5 mm, flip angle = 90°, bandwidth 124 Hz/Px, a 2D TI scout planned perpendicular to the largest diameter of the aorta (matrix = 576, FOV = 340 mm, slice thickness = 0.6 mm, TR between subsequent IR pulses = 1,000 ms, TR = 44.91, TE = 2.09 ms, spatial resolution = 0.6 × 0.6 × 3 mm, flip angle = 35°, bandwidth 579 Hz/Px, a T2*-weighted sequence (matrix = 832, FOV = 150 mm, slice thickness = 0.3 mm, TR = 15.0 ms, TE = 4.0 ms, TR between subsequent IR pulses = 1,000 ms, spatial resolution = 0.18 mm and flip angle = 20°) and finally a high-resolution inversion recovery sequence for visualization of the Gd-based collagen-specific probe (matrix = 384, FOV = 57 mm, 56 slices, slice thickness = 0.3 mm, TR = 1,019,72 ms, TE = 7,02 ms, TR between subsequent IR pulses = 1,000 ms, spatial resolution = 0.1 × 0.1 × 0.3 mm, flip angle = 30°, bandwidth 130 Hz/px). Our delayed enhancement IR scans were preceded by a 2D Look-Locker sequence planned perpendicular to the ascending aorta, which was then used to determine the optimal inversion time (TI) for blood signal nulling.

For further analysis, images were analyzed with Visage (Version7.1, Visage Imaging, Germany). To measure the areas of signal enhancement [signal intensity (SI)] within the aneurysmal wall, 2D regions of interests (ROIs) were drawn around the respective areas in native and delayed enhancement inversion recovery MR images, anatomically co-localized with MR angiography images. Three measurements were performed in both the native and delayed enhancement inversion recovery sequences for each aneurysm. First, we visually determined both the beginning and end of the aneurysm and took a first measurement in the middle of each aneurysm. The next two measurements were then taken in the middle of the upper half and in the middle of the lower half of the aneurysm. Finally, average values were calculated from the three measurements. For the T2* signal voids, the size and the signal reduction of the largest signal void (in mm^2^) was determined and extracted for later analysis.

For analysis of the collagen-specific probe, contrast-to-noise-ratios (CNR) were calculated according to the following formula, with noise being defined as the standard deviation in the background ROI placed in the air anterior to the aorta: CNR = (SI_aneurysmal wall_ − SI_blood_)/SD_air_.

### Collagen-specific probe

EP-3533 was used as a collagen-specific probe^32,33^. The probe shows a good affinity for collagen type 1 (KD = 1.8 ± 1.0 µM) with a relaxivity of 16.1 mM^−1^·s^−1^ per Gd^3+^ (or 48.3 mM^−1^·s^−1^ per molecule) at a field strength of 1.41 T and 25 °C (PBS), while the gadolinium moieties generate a strong T1 signal^32,34^. The blood half-life of EP-3533 in mice was previously determined as 19 min and it was previously tested at 10 µM without showing a measurable effect in terms of inhibiting receptor binding or enzymatic activity^35^. Caravan et el. showed that could be used for molecular imaging of collagen in the context of fibrosis and extracellular matrix remodeling^19,32^.

### Iron-oxide-based inflammation-specific probe

Iron oxide particles can be used for molecular, cellular and vascular imaging^36^. Ferumoxytol consists of ultra-small superparamagnetic particles of iron oxide (USPIO) and received approval of the Food and Drug Administration (FDA) in 2009 for the treatment of iron deficiency anemia in adult chronic kidney disease patients^37^. Ferumoxytol consists of multidisperse nanoparticles with a Fe_3_O_4_ core, a carbohydrate coating, a hydrodynamic diameter of 30 nm and a long blood half-life of approximately 14 h^37^. Therefore, in the present study, MR imaging was performed 24 h after administration of a clinical dose of ferumoxytol (Feraheme, AMAG Pharmaceuticals, Waltham, MA, USA) via the tail vein (4 mg Fe/kg). Ferumoxytol nanoparticles have a r_1_ relaxivity of 15 s^−1^ mm^−1^ and a r_2_ relaxivity of 89 s^−1^ mm^−1^ (1.5 T, 37 °C)^38^. Due to its strong T2 and T2* shortening effects with a resulting signal void in the corresponding MR images, it is considered a negative contrast agent. After intravenous application, USPIO accumulate in macrophages, whereby several mechanisms have been proposed, such as USPIO being endocytosed by activated blood monocytes, transcytosis of USPIO followed by progressive endocytosis by in situ macrophages and transport of USPIO into the pathological tissue^38^.

### Competition experiments

Four weeks after the implantation of the osmotic minipumps, in vivo competition experiments were performed (n = 3), whereby all imaging was performed within a 48-h imaging session. On day 1, animals were imaged before and after the administration of the collagen-specific probe. On day 2 (after 24 h), imaging was performed before and after administration of a tenfold higher dose of a nonparamagnetic collagen peptide. The collagen-specific probe was then administered at a clinical dose of 10 µmol/kg and inversion recovery T1-weighted MRI was performed 30 min after this.

### Histological analysis and immunohistology

After anesthesia and euthanasia by cervical dislocation, terminal exsanguination was performed by perfusion through the left ventricle with physiological saline for 10 min. Subsequently, the abdominal aorta was removed and embedded in a tissue freezing medium (Tissue-Tek O.C.T. compound, Sakura Finetek, Torrance, CA, USA) for later cryosectioning. Aortic tissues were cut at − 20 °C into 7 µm cryosections and then mounted on adhesion slides (SuperFrost, Thermo-Scientific). Cryosections (7 µm) were first stained for Miller’s Elastica van Gieson (EvG) to visualize elastic fibers, for Perls’ Prussian blue stain to visualize the iron ions as a surrogate marker of inflammation and for Picrosirius red and Masson’s trichrome stain for visualization of collagen fibers. The stained histological slices were examined and photographed using a light microscope (Observer Z1, Carl Zeiss Microscopy GmbH, Jena, Germany), whereby the digitized images of Perls’ Prussian blue, Picrosirius red and Masson’s trichrome stain were used for measurements of the iron and collagen contents of the respective probes. All measurements on the digitized images were performed using the software ImageJ (version 1.52, National Institutes of Health, Bethesda, Maryland, USA)^39^. Vessel lumen, adventitia, media and intima were defined as adventitial area, as described previously^21^. For measurement of the collagen fraction as well as the iron fraction of the respective adventitial area, the percentage of the collagen stain area or iron stain area per adventitial area was determined, using the color profile of collagen fibers as the threshold for automatic segmentation.

Immunohistochemistry was performed to show a co-localization of collagen fibers in Picrosirius red and Masson’s trichrome stain with regions of positive immunohistochemical staining. For this, a rabbit polyclonal antibody (Rabbit polyclonal to Collagen I, ab34710, Abcam, Australia, 1:200) was used, which was then incubated with a polyclonal secondary IgG antibody, conjugated to HRP (Goat anti-Rabbit IgG H&L (HRP), ab205718, Abcam, Australia, 1:500) (also refer to Fig. S6).

### Inductively coupled plasma–mass spectrometry

ICP-MS measurements were performed in 12 mice (n = 3 per time-point for the week-by-week study and n = 3 control mice) in order to determine the absolute gadolinium concentrations per probe. For this, half of the dissecting AAA was snap-frozen in liquid nitrogen and before ICP-MS, samples were digested in 0.75 ml of 65% nitric acid at room temperature overnight, followed by dilution with 12 ml deionized water to achieve an acid concentration between 3.5 and 3.8%.

### Laser ablation–inductively coupled plasma–mass spectrometry

LA-ICP-MS was performed with a LSX-213-G2 + laser system for elemental bioimaging (CETAC Technologies, USA). Quantification and visualization were performed with an in-house-developed software (Robin Schmid, WWU-Münster, Germany). The laser system was equipped with a two volume HelExII cell, which was connected to an ICP-MS spectrometer (ICPMS-2030, Shimadzu, Japan) via Tygon-tubing. Histological samples were then ablated via line-by-line scan using a spot size of 15 µm, a scan speed of 45 µm s^−1^ and 800 mL min^−1^ Helium as carrier gas. The following analysis was conducted in collision gas mode with He as collision gas and 100 ms integration time for the analyzed isotopes ^31^P, ^64^Zn, ^158^Gd and ^160^Gd. For the quantification of Gd, matrix-matched gelatin-based standards were used (10%, w/w), including a blank spiked with different Gd concentrations ranging from 1 to 5.000 µg/g. For the averaged intensities of the scanned lines, there was a good linear correlation with a regression coefficient of R^2^ = 0.9999 within this concentration range. The limits of detection (LOD) and quantification (LOQ) were 8 ng/g and 28 ng/g Gd, respectively, whereby they were calculated with the 3σ- and 10σ-criteria.

### Statistical analysis

The statistical software ‘R’ (Version 3.6.1, R-Development-Core-Team, 2019) was used for statistical analysis^40^. To determine required group sizes, a prior analysis was performed, applying an effect strength of 0.5 (Cohen's d) to the main size, assuming 50% expected difference between test and sham-operated group. The Shapiro–Wilk test was used in order to test for normal distribution of grouped continuous variables. In case of normal distribution of continuous variables, students’ t-test was performed. Variables, which were not normally distributed, were compared using the Mann–Whitney-U test. Univariate calculations were calculated using Pearson’s correlation coefficient. For illustration, box plots or point range charts were used. In the longitudinal study, the risk of rupture was assessed based on a receiver operating characteristic (ROC) analysis. Subsequently, sensitivity and specificity were calculated. Significance levels are indicated as follows: p < 0.05, p < 0.01, p < 0.001, and p < 0.0001.

## Supplementary information

Supplementary Information.

## Data Availability

All data are available in the manuscript and the supporting figures.

## References

[1] Kuhnl A (2017). Incidence, treatment and mortality in patients with abdominal aortic aneurysms. Dtsch. Arztebl. Int..

[2] Didangelos A (2011). Extracellular matrix composition and remodeling in human abdominal aortic aneurysms: a proteomics approach. Mol Cell Proteomics.

[3] Abdul-Hussien H (2007). Collagen degradation in the abdominal aneurysm: a conspiracy of matrix metalloproteinase and cysteine collagenases. Am. J. Pathol..

[4] Sakalihasan N, Limet R, Defawe OD (2005). Abdominal aortic aneurysm. Lancet.

[5] Lutgens SP, Cleutjens KB, Daemen MJ, Heeneman S (2007). Cathepsin cysteine proteases in cardiovascular disease. FASEB J..

[6] Erbel R (2014). 2014 ESC Guidelines on the diagnosis and treatment of aortic diseases: document covering acute and chronic aortic diseases of the thoracic and abdominal aorta of the adult. The Task Force for the Diagnosis and Treatment of Aortic Diseases of the European Society of Cardiology (ESC). Eur. Heart J..

[7] Daugherty A, Cassis LA (2004). Mouse models of abdominal aortic aneurysms. Arterioscler Thromb. Vasc. Biol..

[8] Phillips EH, Lorch AH, Durkes AC, Goergen CJ (2018). Early pathological characterization of murine dissecting abdominal aortic aneurysms. APL Bioeng..

[9] Trachet B (2017). Angiotensin II infusion into ApoE-/- mice: a model for aortic dissection rather than abdominal aortic aneurysm?. Cardiovasc. Res..

[10] Adelsperger AR (2018). Development and growth trends in angiotensin II-induced murine dissecting abdominal aortic aneurysms. Physiol. Rep..

[11] LysgaardPoulsen J, Stubbe J, Lindholt JS (2016). Animal models used to explore abdominal aortic aneurysms: a systematic review. Eur. J. Vasc. Endovasc. Surg..

[12] Ashton HA (2002). The multicentre aneurysm screening study (MASS) into the effect of abdominal aortic aneurysm screening on mortality in men: a randomised controlled trial. Lancet.

[13] Bourrinet P (2006). Preclinical safety and pharmacokinetic profile of ferumoxtran-10, an ultrasmall superparamagnetic iron oxide magnetic resonance contrast agent. Invest. Radiol..

[14] Thompson RW, Geraghty PJ, Lee JK (2002). Abdominal aortic aneurysms: basic mechanisms and clinical implications. Curr. Probl. Surg..

[15] Klink A (2011). In vivo characterization of a new abdominal aortic aneurysm mouse model with conventional and molecular magnetic resonance imaging. J. Am. Coll. Cardiol..

[16] Verollet C (2011). Extracellular proteolysis in macrophage migration: losing grip for a breakthrough. Eur. J. Immunol..

[17] Bierry G (2009). Macrophage imaging by USPIO-enhanced MR for the differentiation of infectious osteomyelitis and aseptic vertebral inflammation. Eur. Radiol..

[18] Polasek M (2012). Molecular MR imaging of liver fibrosis: a feasibility study using rat and mouse models. J. Hepatol..

[19] Zhu B (2017). Combined magnetic resonance elastography and collagen molecular magnetic resonance imaging accurately stage liver fibrosis in a rat model. Hepatology.

[20] Trollope A, Moxon JV, Moran CS, Golledge J (2011). Animal models of abdominal aortic aneurysm and their role in furthering management of human disease. Cardiovasc. Pathol..

[21] Brangsch J (2019). Concurrent molecular magnetic resonance imaging of inflammatory activity and extracellular matrix degradation for the prediction of aneurysm rupture. Circ. Cardiovasc. Imaging.

[22] Investigators MRS (2017). Aortic wall inflammation predicts abdominal aortic aneurysm expansion, rupture, and need for surgical repair. Circulation.

[23] Truijers M (2009). In vivo imaging of the aneurysm wall with MRI and a macrophage-specific contrast agent. AJR Am. J. Roentgenol..

[24] Zheng KH (2019). Plaque permeability assessed with DCE-MRI associates with USPIO uptake in patients with peripheral artery disease. JACC Cardiovasc. Imaging.

[25] Adams LC (2020). Noninvasive imaging of vascular permeability to predict the risk of rupture in abdominal aortic aneurysms using an albumin-binding probe. Sci. Rep..

[26] Messroghli DR (2004). Modified look-locker inversion recovery (MOLLI) for high-resolution T1 mapping of the heart. Magn. Reson. Med..

[27] Saraff K, Babamusta F, Cassis LA, Daugherty A (2003). Aortic dissection precedes formation of aneurysms and atherosclerosis in angiotensin II-infused, apolipoprotein E-deficient mice. Arterioscler Thromb. Vasc. Biol..

[28] Wang YX (2001). Angiotensin II increases urokinase-type plasminogen activator expression and induces aneurysm in the abdominal aorta of apolipoprotein E-deficient mice. Am. J. Pathol..

[29] Halloran BG, Davis VA, McManus BM, Lynch TG, Baxter BT (1995). Localization of aortic disease is associated with intrinsic differences in aortic structure. J. Surg. Res..

[30] Toth GB (2017). Current and potential imaging applications of ferumoxytol for magnetic resonance imaging. Kidney Int..

[31] Daugherty A, Manning MW, Cassis LA (2000). Angiotensin II promotes atherosclerotic lesions and aneurysms in apolipoprotein E-deficient mice. J. Clin. Invest..

[32] Caravan P (2007). Collagen-targeted MRI contrast agent for molecular imaging of fibrosis. Angew Chem. Int. Ed. Engl..

[33] Helm PA (2008). Postinfarction myocardial scarring in mice: molecular MR imaging with use of a collagen-targeting contrast agent. Radiology.

[34] Vithanarachchi SM, Allen MJ (2012). Strategies for target-specific contrast agents for magnetic resonance imaging. Curr. Mol. Imaging.

[35] Fuchs BC (2013). Molecular MRI of collagen to diagnose and stage liver fibrosis. J. Hepatol..

[36] Wang YX, Hussain SM, Krestin GP (2001). Superparamagnetic iron oxide contrast agents: physicochemical characteristics and applications in MR imaging. Eur. Radiol..

[37] Gkagkanasiou M, Ploussi A, Gazouli M, Efstathopoulos EP (2016). USPIO-enhanced MRI neuroimaging: a review. J. Neuroimaging.

[38] Corot C, Robert P, Idee JM, Port M (2006). Recent advances in iron oxide nanocrystal technology for medical imaging. Adv. Drug. Deliv. Rev..

[39] Rasband, W. S. *ImageJ*, U. S. National Institutes of Health, Bethesda, MD, USA, https://imagej.nih.gov/ij/ (1997–2018).

[40] Wickham, H. The Tidyverse. *R package ver. 1.1*, p. **1** (2017).

