# Supplementary material for: Simultaneous molecular MRI of extracellular matrix collagen and inflammatory activity to predict abdominal aortic aneurysm rupture
Source: Sci Rep. 2020 Sep 16;10:15206. doi: 10.1038/s41598-020-71817-x (PMC7494914; doi:10.1038/s41598-020-71817-x)
Supplement: Supplementary file 1 — Supplementary Information. [file 41598_2020_71817_MOESM1_ESM.docx]

**Supporting Information (SI)**

**Title: Simultaneous molecular MRI of extracellular matrix collagen and inflammatory activity to predict abdominal aortic aneurysm rupture**

**Author list: Lisa C. Adams, Julia Brangsch, Carolin Reimann, Jan O. Kaufmann, Rebecca Buchholz, Uwe Karst, Rene M. Botnar, Bernd Hamm, Marcus R. Makowski**

**Figures**


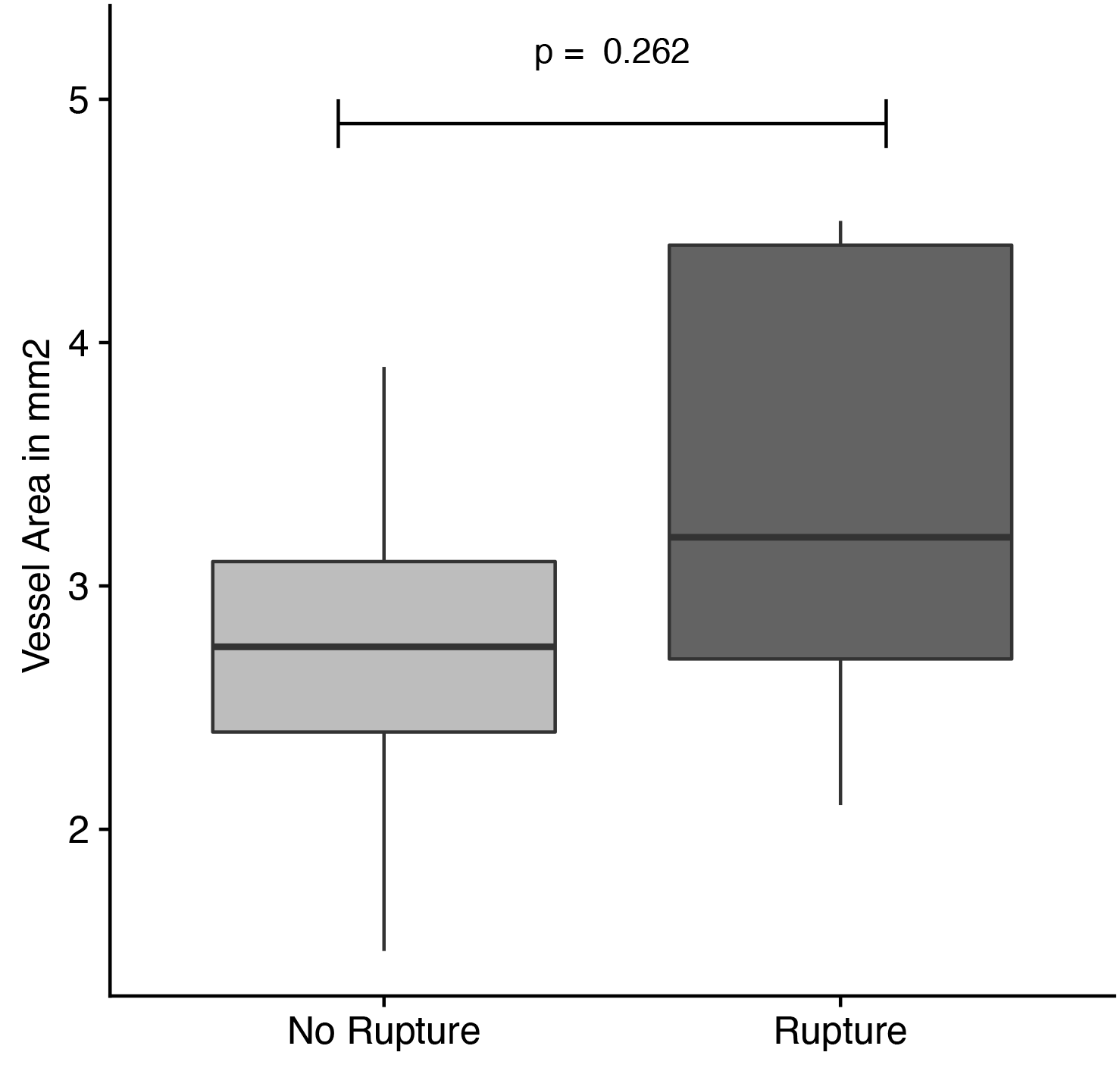


**Figure S1.** Differences in vessel area (mm^2^) between stable and rupturing AAAs. There was no significant difference (p=0.262).

**
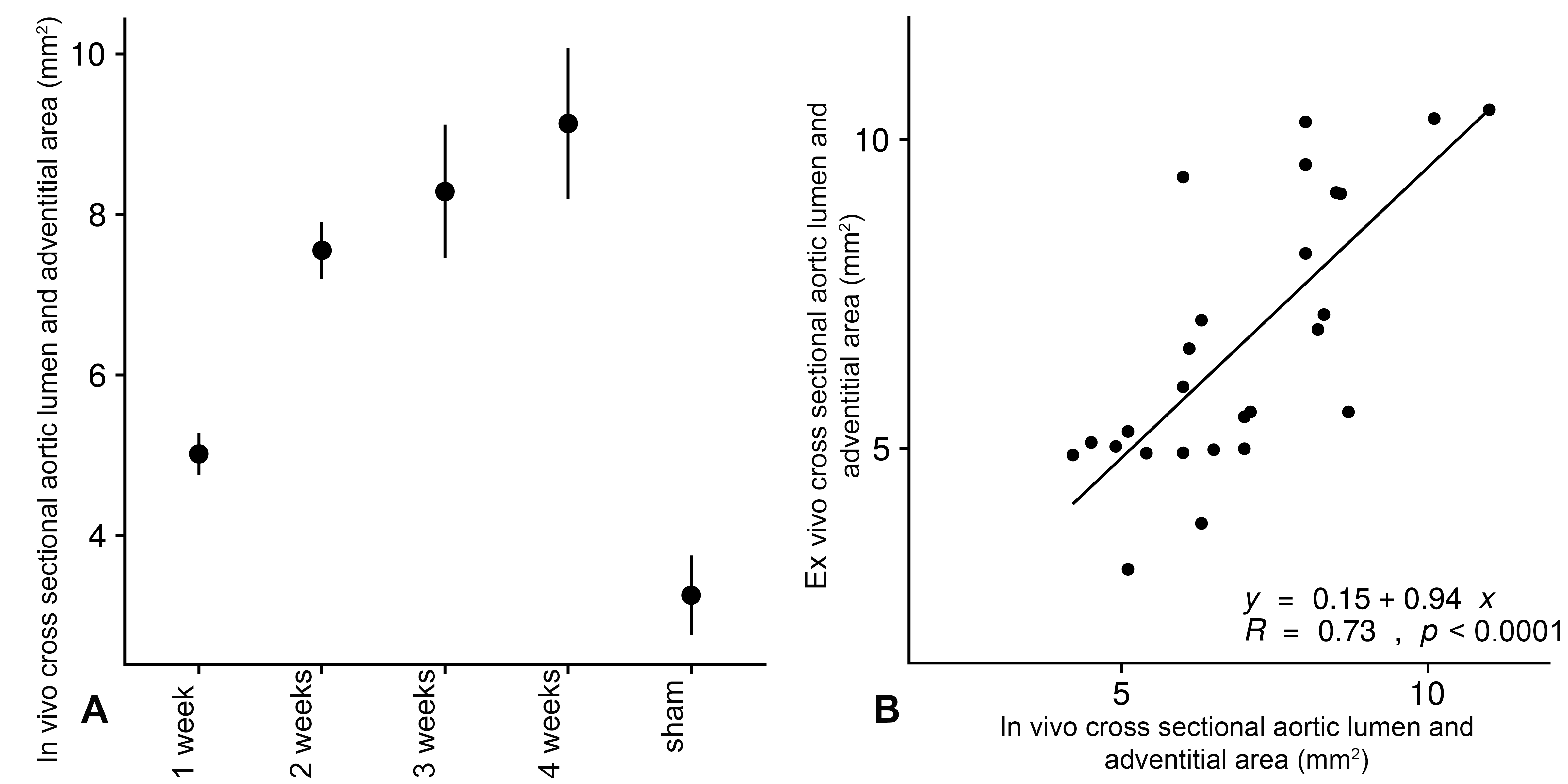
**

**Figure S2**. In vivo and ex vivo measurements of the aneurysmal adventitial area. A) In vivo MR measurements revealed an increase in the aneurysmal adventitial areas (mm2) over the course of four weeks with continuous Angiotensin II release, which can be seen as an indicator for aneurysm development. B) In vivo MR measurements furthermore showed a close correlation with ex vivo measurements on cryosections with a correlation coefficient of R=0.73.

**
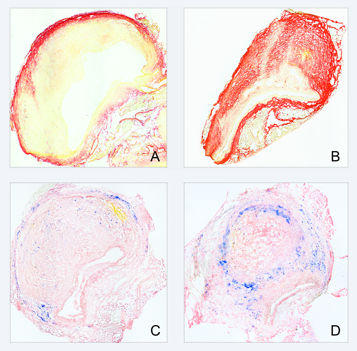
**

**Figure S3.** Different collagen and iron content observed by histology. A, representative image of a collagen-poor four-week old aneurysm. B, representative image of a collagen-rich two-week old aneurysm. This correlated with the different enhancement patterns observed by MRI, also with regard to the longitudinal study. C and D, representative images of a two-week-old aneurysm with comparatively low (C) and a four-week-old aneurysm with high (D) iron content, indicative of macrophage activity and inflammatory processes.

**
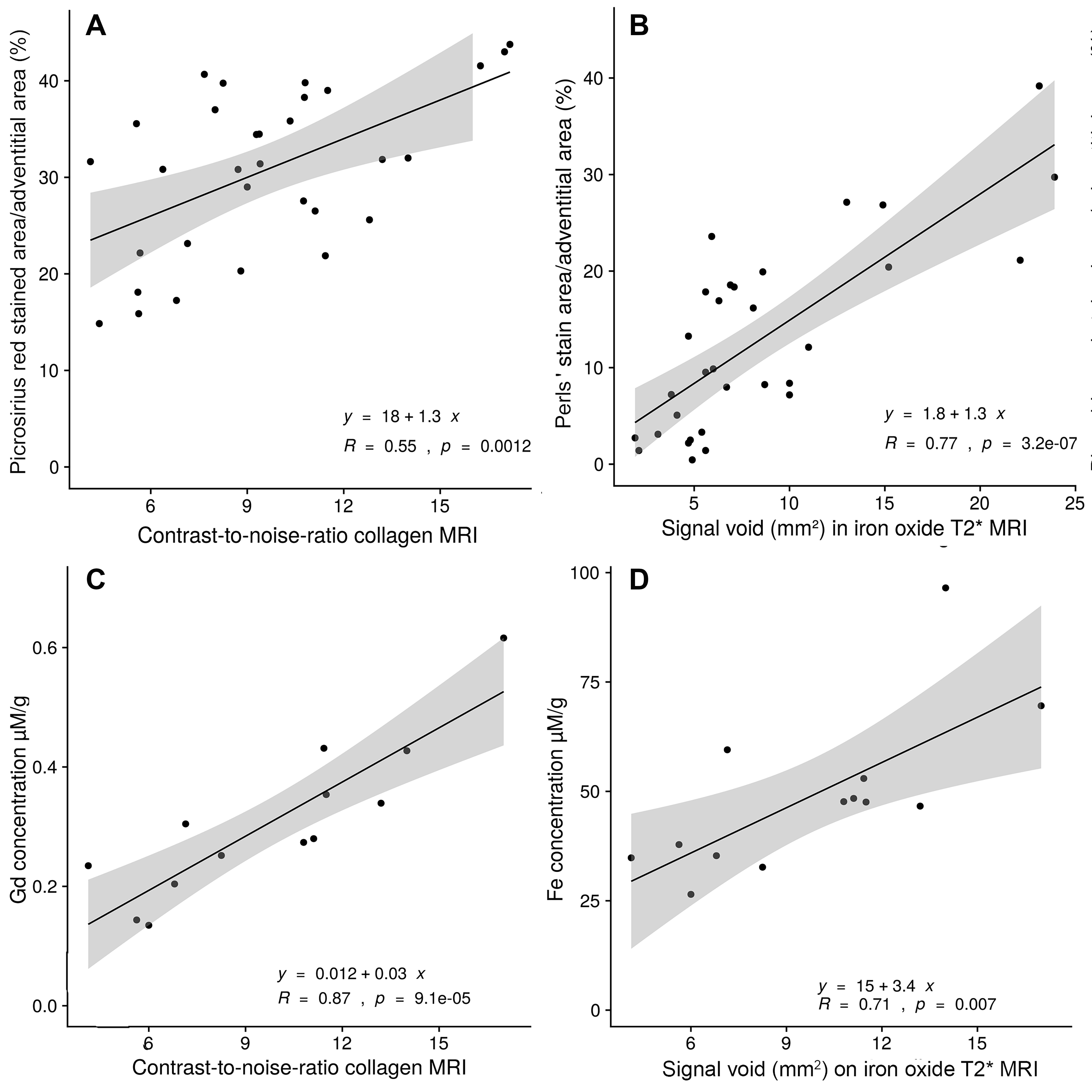
**

**Figure S4.** Correlation of in vivo and ex vivo measurements. *A)* In vivo CNR measurements after injection of the collagen-specific probe showed a moderated correlation with ex vivo histological Picrosirius Red-stained sections. *B)* In vivo measured areas of T2* signal voids (mm^2^) strongly correlated with histological measurements on Perls’ Prussian-Blue-stained sections. *C)* ICP-MS revealed a strong correlation between gadolinium concentration and in vivo CNR measurements after the injection of the collagen-targeted probe. *D)* A strong correlation was also observed between the iron concentration as determined by ICP-MS and the size of the T2* signal voids following the administration of the iron-oxide particles. In summary, these measurements indicate a moderate to strong agreement between *in vivo* and *ex vivo* measurements of the collagen specific probe and the iron oxide particles. *Abbreviations:* CNR: Contrast to noise ratio; ICP-MS: Inductively coupled mass spectroscopy.

**
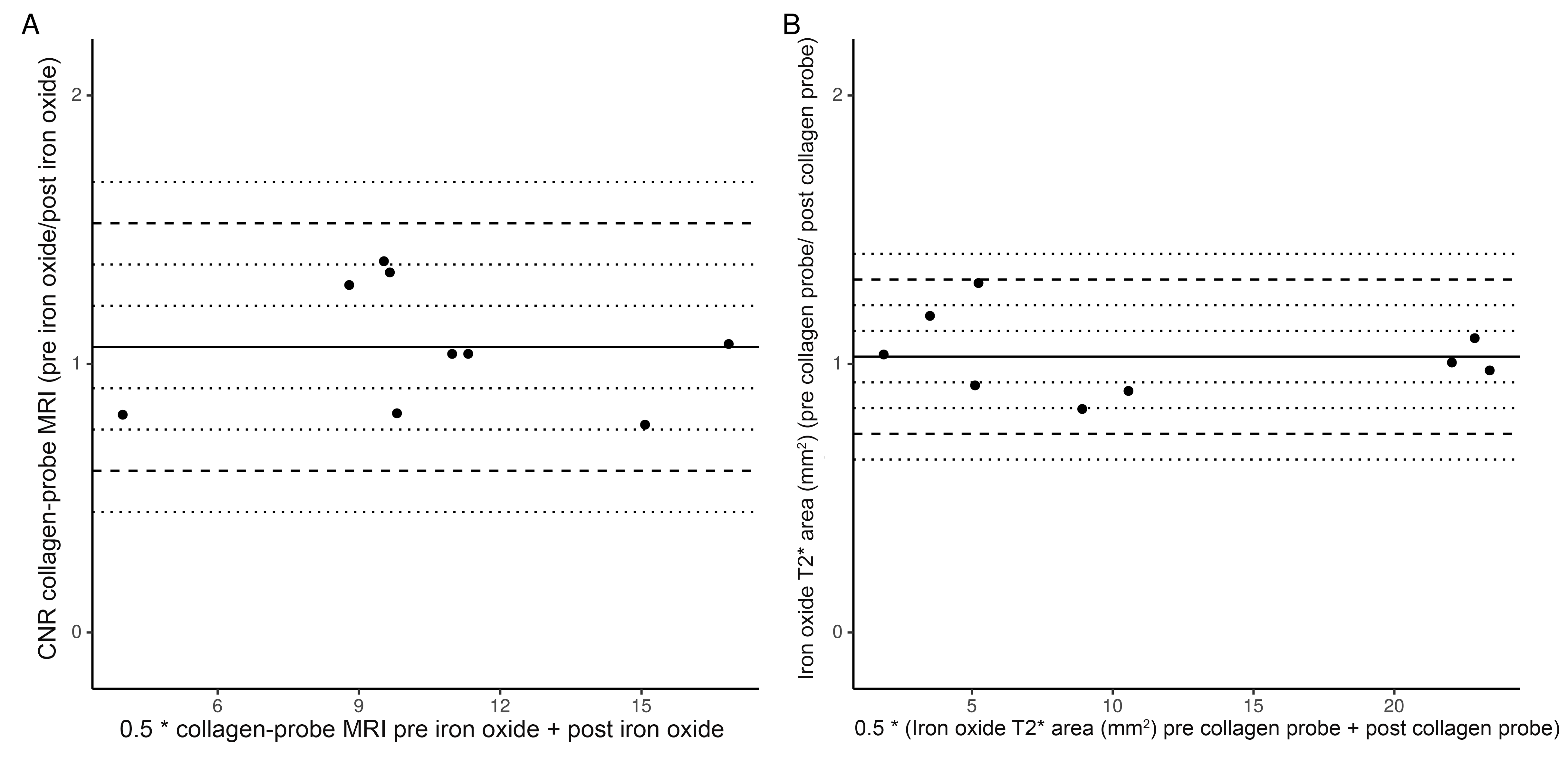
**

**Figure S5.** Effect of both imaging probes on the visualization of each other. *A)* To evaluate the potential influence of iron-oxide particles on the assessment of the collagen-targeted probe, T1-weighted MRI was performed prior and 24 hours after the injection of iron-oxide particles. An excellent correlation of CNR measurements prior to and after the injection of iron-oxide particles could be demonstrated (ICC= 0.99 (95% confidence interval: 0.97-0.99). *B)* T2*-weighted MRI was also performed prior to and after the injection of the collagen-targeted probe in order to investigate a potential effect of the collagen-targeted probe on the evaluation of the iron-oxide particles. These measurements showed a good correlation with an ICC of 0.82 (95% confidence interval: 0.40-0.96). *Abbreviation:* CNR: contrast-to-noise-ratio.

**
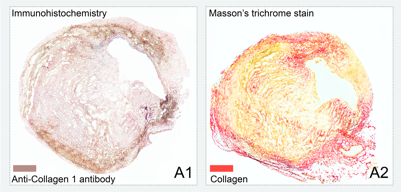
**

**Figure S6.** Ex vivo immunohistochemistry and picrosirius red staining. A1, anti-collagen-1-antibody staining and B1, picrosirius red staining for visualization of collagen fibers, confirming the co-localization of collagen-1-specific immunohistochemistry with picrosirius red staining. Areas positive for collagen 1 in immunohistochemistry correspond well to the red areas highlighted by Picrosirius red staining.
